# Neuroprotective Natural Molecules, From Food to Brain

**DOI:** 10.3389/fnins.2018.00721

**Published:** 2018-10-23

**Authors:** Joaquin González-Fuentes, Jorge Selva, Carmen Moya, Lucia Castro-Vázquez, Maria V. Lozano, Pilar Marcos, Maria Plaza-Oliver, Virginia Rodríguez-Robledo, Manuel J. Santander-Ortega, Noemi Villaseca-González, Maria M. Arroyo-Jimenez

**Affiliations:** Cellular Neuroanatomy and Molecular Chemistry of Central Nervous System, Faculty of Pharmacy and Faculty of Medicine, University of Castilla-La Mancha, CRIB (Regional Centre of Biomedical Research), Albacete, Spain

**Keywords:** vitamin E, vitamin C (ascorbic acid), nanoplatforms, analytical – methods, aging, neuroprotective effect

## Abstract

The prevalence of neurodegenerative disorders is increasing; however, an effective neuroprotective treatment is still remaining. Nutrition plays an important role in neuroprotection as recently shown by epidemiological and biochemical studies which identified food components as promising therapeutic agents. Neuroprotection includes mechanisms such as activation of specific receptors, changes in enzymatic neuronal activity, and synthesis and secretion of different bioactive molecules. All these mechanisms are focused on preventing neuronal damage and alleviating the consequences of massive cell loss. Some neuropathological disorders selectively affect to particular neuronal populations, thus is important to know their neurochemical and anatomical properties in order to design effective therapies. Although the design of such treatments would be specific to neuronal groups sensible to damage, the effect would have an impact in the whole nervous system. The difficult overcoming of the blood brain barrier has hampered the development of efficient therapies for prevention or protection. This structure is a physical, enzymatic, and influx barrier that efficiently protects the brain from exogenous molecules. Therefore, the development of new strategies, like nanocarriers, that help to promote the access of neuroprotective molecules to the brain, is needed for providing more effective therapies for the disorders of the central nervous system (CNS). In order both to trace the success of these nanoplatforms on the release of the bioactive cargo in the CNS and determinate the concentration at trace levels of targets biomolecules by analytical chemistry and concretely separation instrumental techniques, constitute an essential tool. Currently, these techniques are used for the determination and identification of natural neuroprotective molecules in complex matrixes at different concentration levels. Separation techniques such as chromatography and capillary electrophoresis (CE), using optical and/or mass spectrometry (MS) detectors, provide multiples combinations for the quantitative and qualitative analysis at basal levels or higher concentrations of bioactive analytes in biological samples. Bearing this in mind, the development of food neuroprotective molecules as brain therapeutic agents is a complex task that requires the intimate collaboration and engagement of different disciplines for a successful outcome. In this sense, this work reviews the new advances achieved in the area toward a better understanding of the current state of the art and highlights promising approaches for brain neuroprotection.

## Aging, Cognitive Decline, and Dietary Antioxidants

Brain aging is a highly complex biological process characterized by a progressive decline of cognitive and physiological abilities. Episodic, working, and spatial memories, as well as processing speed, decline throughout physiological brain aging (Hedden and Gabrieli, 2004). Motor and sensory functions are also sensitive to the aging process (Smith et al., 1999; Liu and Yan, 2007), and one of the biggest neurobiological challenges in the last decades has been the knowledge of age-associated changes in order to delay cognitive senescence.

Several causal mechanisms of brain aging have been proposed (Yeoman et al., 2012), and one of the most relevant is oxidative stress (OS), caused by an imbalance between generation and detoxification of reactive oxygen species (ROS). The free radical theory of aging postulated by Harman in 1956 points that excessive production and accumulation of ROS causes a subsequent altered cellular integrity that eventually leads to severe and irreversible damage (Harman, 1956; Liochev, 2013), thus OS plays a crucial role in neuronal damage associated with aging as well as with neurological disorders (Vina et al., 2013).

Supplementation with dietary antioxidants can alleviate the redox imbalance. Two important key issues have to be keep in mind regarding the dietary supplementation. The first is the bioavailability (F) of these molecules, their ability to cross membranes and reach the tissues in an appropriated amount to exert their effect. The second aspect is the special characteristics of the biological samples (complex matrix, low volume or weight and trace levels of the targets), which determines that the right choice of the methods of analysis will be essential to ensure a correct interpretation of the results.

The administration of antioxidant molecules extracted from natural sources has been proposed as an alternative form of treatment of age-associated decline in normal brain function (Magrone et al., 2012; Dacks et al., 2013; Prakash and Kumar, 2013), and in this sense vitamins have been used widely as antioxidant therapy. The brain levels of some neurotransmitters have been tested before and after the administration of a single vitamin, and it has been reported that VitD or VitA could have an impact on the levels of some neurotransmitters (Orme et al., 2016; Pertile et al., 2016; Guo et al., 2018; Luan et al., 2018). However, the most important vitamins regarding their role in the OS reduction and prevention against age-related neurodegeneration are VitC and VitE (Ramis et al., 2016; Sun et al., 2018).

Here, we review the literature that explores the potential of these natural antioxidant vitamins to protect the brain against the aging process. We focus in their effects, the problems and solutions to improve their F, and the most suitable techniques to analyse their levels in biological samples.

## Vitamins and Oxidative Stress

The most powerful water-soluble antioxidant in the organism is VitC, present physiologically as ascorbate anion (Rice, 2000; Harrison et al., 2014). Mammals can synthetize VitC in the liver, with the exception of humans, primates or guinea pigs that need to consume it from the diet. In all the cases, ascorbate passes from cerebrospinal fluid to deep brain structures by diffusion, and a sodium-dependent transporter (SVCT2) concentrates ascorbate intracellularly (Rebec and Pierce, 1994; Rice, 2000). The most important neuroprotective action of ascorbate is exerted by regulation of extracellular glutamate levels. Excessive glutamate release and accumulation produces neurotoxicity (Rebec et al., 2003), and the activation of extracellular glutamate uptake involves the release of ascorbate to the extracellular medium by a glutamate-ascorbate heteroexchange membrane transporter (Rice, 2000). The extracellular concentration of ascorbate in brain tissue is maintained homeostatically at the expense of intracellular stores (Rebec and Pierce, 1994; Qiu et al., 2007), and ascorbate may also offer protection at the intracellular compartment (Ballaz et al., 2013).

VitE is the most effective chain-breaking, lipid-soluble antioxidant in cellular membranes (Mocchegiani et al., 2014), and is one of the major scavengers of radical-oxygenated species in nervous cells (Crouzin et al., 2010). It traps free radicals and breaks the chain reaction, preventing the propagation of lipid peroxidation. This reaction produces a tocopheroxyl radical, which requires ascorbate for its regeneration back to reduced VitE (Mocchegiani et al., 2014; Dolu et al., 2015). Thus, the antioxidant effect of VitE is potentiated by co-administration with VitC. In fact, previous studies carried out in animal models (Harrison and May, 2009) and in humans (Kontush et al., 2001) reported a more powerful neuroprotective effect when the two vitamins are administered together.

VitE is taken from the diet, incorporated into lipoproteins, and delivered systemically (Mocchegiani et al., 2014). Such distribution is possible due to the α-tocopherol transfer protein (α-TTP), which controls the hepatic uptake of VitE. α-TTP is present in many organs, including the brain (Manor and Morley, 2007), but its effect on VitE transport remains unclear.

Thus, VitC and VitE are transported into neurons by different carrier proteins, and accumulated by separate systems that act synergically (Spector and Johanson, 2007). A recent work (Marcos et al., 2018) reports the brain distribution of SVCT2 and α-TTP, which display specific patterns that remain unchanged with age. Besides, they are present mainly in neurons but not in astrocytes, and this could contribute to explain the selective responses observed in neurons against OS (Wang and Michaelis, 2010).

VitC and VitE have been successfully tested in several *in vitro* and in animals models studies in order to improve aging-related process (Santos et al., 2008; Ballaz et al., 2013; Aumailley et al., 2016; Ramis et al., 2016; Sun et al., 2018). However, the results obtained from human trials are not always consistent. Low levels of VitC and VitE, as well as other antioxidants, have been observed in plasma of individuals with Alzheimer’s disease and mild cognitive impairment (Rinaldi et al., 2003; Mangialasche et al., 2012), which has led to the suggestion that supplementation with antioxidants could delay or reduce cognitive impairment. The results of the several trials that have already been carried out in the last decades failed to reach a consensus by the role of these vitamins in the treatment of aging and related disease (Petersen et al., 2005; Goodman et al., 2011; Santilli et al., 2015; Basambombo et al., 2017; Monacelli et al., 2017; Ohlow et al., 2017). This can be due, at least in part, to the heterogeneity (e.g., genetic variations as well as differences in diet, lifestyle and environmental factors) of the human population and the difficulty in finding true controls (Mocchegiani et al., 2014), as well as the inherent variability in amounts of VitE present in regular diets.

## Methodological Approaches to Study

### Nanotechnology

**FIGURE 1:**
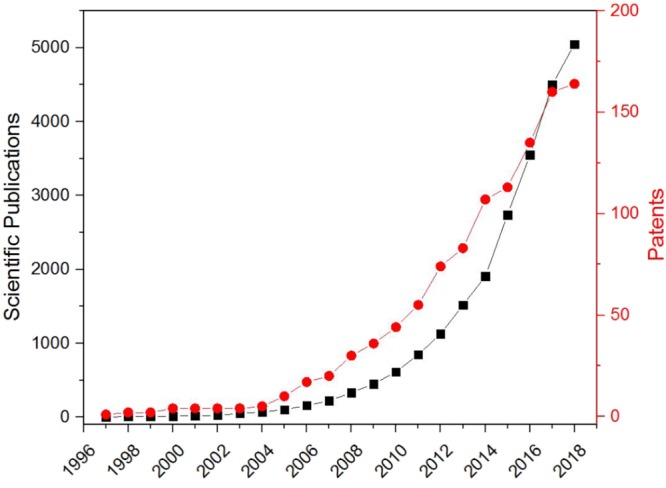
Accumulative number of articles (Scopus) and world patents (WIPO) using the keywords “food” and “nanoparticles” up to June 2018.

Nanotechnology has contributed to food technology with a wide variety of nanoplatforms such as micelles, micro- nano-emulsions, emulsions or nanoparticles (**Figure 1**) (Porter et al., 2007; Cerqueira et al., 2013; Livney, 2015; Yao M. et al., 2015). After oral intake, these nanostructures will be able to improve the F of nutraceuticals enhancing their efficacy. Nanoparticles will be able to: (i) avoid the premature degradation of the molecules in physiological fluids; (ii) improve the solubility of the molecules; (iii) promote the diffusion of the cargo across the blanket of mucus that protects the intestinal tract; (iv) facilitate the intra- or para-cellular internalization of the intact form of the bioactive molecule across the intestinal epithelium, and (v) enable their transport through the blood brain barrier (O’Driscoll and Griffin, 2008; Yang et al., 2011; Ensign et al., 2012; McClements, 2015).

Nanostructures applied in food technology can be classified either as lipid-based or non-lipid-based systems. These nanostructures have been able to improve the F of a wide range of nutraceuticals, such as ω-3/ω-6, PUFAS, polyphenols, vitamins (E, D,…), carotenoids,… (Livney, 2015; Yao M. et al., 2015). Among others, β-carotene is a clear example of the success of this technology. This antioxidant presents a minimal F, being even lower when the natural extract is ingested without food (Faulks and Southon, 2005; Ribeiro et al., 2008). However, by encapsulating this molecule in lipid-based nanoparticles its F can be significantly increased (Acosta, 2009). Similarly, encapsulation of curcumin (Maheshwari et al., 2006) in liposomes or polyester nanoparticles has reduced the intestinal absorption time (Takahashi et al., 2009; Anand et al., 2010). Besides, encapsulation of curcumin significantly improved the F of this polyphenol from 5.3 mg min/L_plasma_ (free curcumin) up to 26.5 mg min/L_plasma_ (encapsulated curcumin). Interestingly, encapsulation did not only increased the plasmatic concentration of curcumin after oral intake, but also preserved better its antioxidant activity along the gastrointestinal tract (GIT) (2.7 folds higher antioxidant activity after oral intake in comparison with the free curcumin) (Takahashi et al., 2009; Anand et al., 2010). Irruption of nanotechnology in food technology has implicated the design of nanoparticles using 100% food grade materials, which has challenged the formulation process (Santiago and Castro, 2016). Besides, the low added value of food supplements has forced to researchers to the use of low cost formulation process (Desai and Park, 2005; Acosta, 2009; Huang, 2012; McClements, 2012).

From a therapeutic point of view, the principal function of nanostructures in food technology relies on their capacity to improve the F of the encapsulated bioactive molecule, preserving its therapeutic activity from the manufacturing of the supplemented food or beverage up to its delivery in systemic circulation. F of the encapsulated molecule can be expressed as a function of the capacity of the nanostructure to overcome the different biological barriers of the oral route as follows (Porter et al., 2007; McClements and Xiao, 2012; Yao et al., 2014; Yao F. et al., 2015):

$$\begin{array}{c} {F=F}_{M}*F_{B}*F_{A} \end{array}$$

Where F_M_ is the fraction of molecule that is not metabolized along the GIT. As will be discussed in more detail below, the fine tune up of the surface properties of the nanostructure will ensure high F_M_ values along the GIT (Plaza-Oliver et al., 2015). F_B_ is the fraction of bioactive molecule bioaccessible, that is, ready to be absorbed through the intestinal cellular wall. Nanostructures can increase the bioaccessibility of the encapsulated molecule through the design different types of nanostructures: mucoadhesive, mucodiffusive or lipid-based digestible. Finally, F_A_ is the fraction of molecule that reach systemic circulation.

Although different manufacturing steps as well as the pass through the GIT does not totally degrade the bioactive molecules, these processes can compromise the activity (Takahashi et al., 2009; Anand et al., 2010). Besides, for some bioactive molecules, such as VitE, it is not enough to reach high F values to ensure the desired therapeutic effect. The different isoforms of VitE (α-, β-, γ-, and δ-tocopherols and the corresponding tocotrienols) present different activities, being the most active isoform the RRR- α-T (Traber and Atkinson, 2007; Traber, 2014). Therefore, it will be necessary to consider not only the total amount of bioactive molecule that reaches systemic circulation, but also the role of these factors on the overall therapeutic effect (T_E_) of a bioactive molecule:

$$\begin{array}{c} T_{E}=F*B_{A} \end{array}$$

Where F represents the overall bioavailability and B_A_ the specific biological activity in plasma. Therefore, to improve the bioavailability or the therapeutic effect of the associated bioactive molecule nanoparticles should be designed in order to enhance F_M_, F_B_, and F_A_. Additionally, the designed nanostructure should preserve as much as possible the biological activity of the associated neuroprotective antioxidant up to reach systemic circulation.

## Instrumental Analytical Chemistry

Despite hybrid analytical techniques result in novel solutions to bioanalytical problems, clinical, medicine or biochemistry fields, are not still used for routine analysis. Hybrid separation techniques are based on couplings between, at least, two instrumental techniques with an appropriate interface, in order to obtain the advantages of the both separation analytical techniques and coupled detector (Careri et al., 1996). Different coupling of liquid or gas chromatography (LC or GC) and CE with sensitive and/or selective detector such as spectroscopy (UV-vis, fluorescence, IR..), electrochemical (EC) and MS (Robledo and Smyth, 2014) are available. Currently, MS has achieved a key role in modern analytical technology due mainly to the online coupling with important separation techniques such as GC (GC-MS), LC (LC-MS/MS), and CE (CE-MS) (Rodríguez Robledo and Smyth, 2009). The extensive development of the last generation of mass spectrometers coupled to highly efficient LC systems has brought better resolution, sensitivity, and reproducibility in a relatively short time-frame (Rodríguez-Suárez et al., 2014). CE is an important, versatile and rapid technique for the separation of a large number of charged analytes, obtaining high separation efficiency in small sample size and low solvent consumption (Rodríguez Robledo and Smyth, 2009). The type of sample, target/s compound/s in the determination or required analysis information, in order to select the most suitable hybrid separation technique, should be considered. In this section, a description and discussion about significant and updated analytical methodology developed for the determination of neuroprotectives molecules, mainly VitC and VitE in complex samples is proposed.

Vitamins can be classified into fat-soluble vitamins (FSVs); and water-soluble vitamins (WSVs). Several methods using separation analytical techniques coupling MS, are available to determine FSVs and WSVs compounds in complex matrices (Phinney et al., 2011; Santos et al., 2012). GC-MS is currently used to determinate FSVs and their metabolites, after solvent extraction and derivatization reaction in human serum or plasma (Coldwell et al., 1987). However, High Performance LC with ultraviolet photometric detection (HPLC-UV) (Bilodeau et al., 2011) and LC-MS/MS are currently preferred in biological matrices (Andreoli et al., 2003; Forchielli et al., 2016).

VitC is highly instable in biological samples. In that way, Kand’ar et al. (Kand’ar and Zakova, 2008) developed a method for the determination of VitC using HPLC-UV to study the stability and recovery into protein precipitation process. Different precipitant agents were tested, and for example, perchloric acid for VitC was unsuitable; however, this HPLC method is a highly sensitive and reproducible for the determination of VitC in human plasma.

Simultaneous determination of L-ascorbic acid using ion-pairing RP-HPLC coupled with EC detector, and other compounds as aminothiols and methionine in biological samples have been presented by Khan et al. (2015). Chromatography/ EC method was applied to determinate reduced forms of the analytes in less than 20 min using as the internal standard *n*-acetyl cysteine.

Since L-ascorbic acid is an electro-active molecule, voltammetric techniques, as EC methodology, are selected for the identification and determination of trace concentration in biological samples (Behfar et al., 2010). A novel voltammetric sensor for determination of VitC based in a moleculary imprinted copolymer has been presented by Kong et al. (2014). The molecularly imprinted copolymer sensor exhibited a high sensitivity, selectivity, good reproducibility and stability for the determination of VitC and comparing to conventional polyaniline-based sensors. In the same way, Wang et al. (2013) developed a simple fluorescence sensor for detection of VitC thanks to the oxidation state change of Au nanoclusters which is controlled by our analyte. The sensor is based in a sensitive turn-off fluorescence a detection limit as low as 0.2 muM applied to biological samples.

Many papers have been published for the simultaneous determination of tocopherols in biological samples (Cervinkova et al., 2016). In the same way, chromatography analytical techniques (GC-MS and LC-MS) for the simultaneous determination of tocopherols and tocotrienols in biological and food matrices have been published by Bartosinska et al. (2016).

Simultaneous determination of VitE and other FSVs and their metabolites in serum of children with cystic fibrosis. They revealed significant changes in the plasma level of the analyzed compounds, VitE among them, presents at extremely low concentrations in patients in comparison to healthy controls (Konieczna et al., 2016).

Simultaneous determination of VitA, VitD, and VitE (Albahrani et al., 2016) and their metabolites have been described using LC-MS/MS in biological samples (Jiang et al., 2015).

A paper comparing two MS methods using chromatography- MS and LC-MS was published by Mottier et al. (2002) for the quantification of α-T and its oxidation product α-tocopherolquinone (TQ) in human plasma. Method validations were carried out in plasma of male volunteer pre- and post-exercise. Both techniques showed that the ratio of TQ/α-T was elevated by 35% immediately after exercise and had returned to basal levels 24 h later.

In cultured cell lines, tissue, plasma and red blood cells, in human or mouse samples, a single HPLC method has been also developed and validated for determination of all levels using a single protocol of extraction of different types of samples (Cimadevilla et al., 2015). Other singular sample was used for Kandar et al. (2014) for determination of retinol and α-T using an HPLC with UV detection in human seminal plasma.

Some analytical methods have been developed for determination of the last eight isomers in biological samples (Liang et al., 2013). This method was used for carrying out pharmacokinetic studies. Small volume of plasma were extracted with an average recovery of 44.7% and an average matrix effect of -2.9%.

Determination of α and δ-tocopherol (δ-T) in plasma, liver and brain samples after high dietary supplementation was developed by Baxter et al. (2012). This study provided further information on their *in vivo* functions and pharmacological effects since their pharmacokinetic properties remain poorly characterized. These results show that high dosage α-T and δ-T in mouse and supplementation of sesamin with δ-T further increases δ-T levels over those seen with δ-T supplementation alone.

An ultrafast analytical methodology for determination of α-T in different biopharmaceutical samples using LC-diode array detector on-line ESI–MS/MS has been currently published (Villaseca-Gonzalez et al., 2018). The optimized and validated chromatographic method is presented as valuable analytical tool for the determination of α-T in loaded drug delivery systems in blood samples.

## Conclusion

In sum, many natural compounds have shown to interact with chemical reactions such as the OS, lipid peroxidation, apoptosis and other mechanisms involved in neuronal damage. In addition, those molecules are able to exert different actions on the neurotransmitter systems. However, nanoplatforms, that improve the F and efficacy of the biomolecules, and correct analytical techniques, are essentials tools in order to obtain the desired results of the treatment to prevent age-related cognitive, motor and sensory impairment.

## Author Contributions

JS, NV-G, MP-O, and CM acquired, analyzed, and interpreted the data. ML and MS-O drafted and revised the work, and designed, acquired, analyzed, and interpreted the data related to pharmaceutical technologies. LC-V and VR-R drafted and revised the work, and designed, acquired, analyzed, and interpreted the data related to chemical analysis and nutrition. JG-F, PM, and MA-J drafted the work and revised it critically for important intellectual content, and designed, acquired, analyzed, and interpreted the data related to neuroscience. All authors approved the final version of the manuscript to be published and agreed to be accountable for all aspects of the work in ensuring that questions related to the accuracy or integrity of any part of the work are appropriately investigated and resolved.

## Conflict of Interest Statement

The authors declare that the research was conducted in the absence of any commercial or financial relationships that could be construed as a potential conflict of interest.
